# Right subclavian artery cannulation: Is chest roentgenogram sufficient to diagnose the complication?

**DOI:** 10.4103/0972-5229.78234

**Published:** 2011

**Authors:** Amit Jain

**Affiliations:** Department of Anaesthesia & Intensive Care, Alchemist Hospitals Ltd., Panchkula, Haryana, India

Dear Editor,

I read with interest the letter to the editor, “Finding on a chest radiograph: A dangerous complication of subclavian vein cannulation” by Srinivasan and Kumar.[1] The inference of the authors seems to be simple, and is based on the prior, yet limited, reports of the radiographic findings of inadvertent subclavian artery cannulation and interpretation of the anatomy of the great vessels of body.[2,3] However, a closer look reveals the omission of many simple and easily available methods that should have been used to further confirm the diagnosis before abruptly removing the catheter in the hemodynamically unstable patient.

Ultrasound-guided insertion of central venous cannulation and trans-thoracic or trans-esophageal Doppler, when available, are the most reliable techniques to diagnose subclavian artery cannulation. However, these may not be readily available and need expertise. Pressure tracings using pressure transducer can also differentiate between venous or arterial cannulation.[3] However, when not available, various alternate bedside techniques should be used in addition to chest roentgenogram for confirming the subclavian artery malpositioning of central venous catheter.

Absence of free flow of intravenous fluid may be possible even if the catheter tip is in the lumen of subclavian vein with the tip abutting walls of the vein. Further, pulsatile movements of the fluid column (at a rate similar to the patient’s heart rate) should appear if the catheter is in artery, at least when the pulse pressure is 40 mmHg, i.e. ≈55 cm of water (as the patient’s blood pressure was 80/40 mmHg). Blood gas analysis of the samples aspirated from the central venous catheter lumen and from the peripheral artery (e.g. radial artery) can be compared in such confusing situations. This method could be a safe, easy and reliable method to diagnose an inadvertent arterial cannulation, especially in intensive care unit settings.

Nonetheless, the post procedural chest radiographic findings of subclavian artery cannulation are important to understand. The usefulness of the post procedural chest radiograph is increased by the fact that even with the electrocardiogram monitoring of the tip, using either electrolyte solution or wire stylet, all catheters–arterial and venous–could reveal an increase in size of the P wave as well as the QRS complex, once the catheter tip extends beyond the pericardial reflection.[4]
